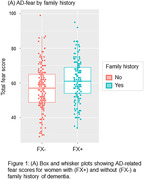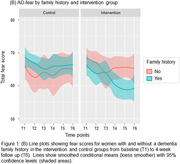# Fear of Alzheimer’s disease in older women with a family history of dementia and what we can do about it: results from an observational study and randomized controlled trial

**DOI:** 10.1002/alz.090823

**Published:** 2025-01-09

**Authors:** Francesca R Farina, Marc P Bennett, James W Griffith

**Affiliations:** ^1^ Global Brain Health Institute, Trinity College Dublin, Dublin Ireland; ^2^ Northwestern University, Chicago, IL USA; ^3^ University College Dublin, Dublin, Dublin Ireland

## Abstract

**Background:**

Alzheimer’s disease (AD) is highly feared. Fear can prevent individuals from seeking help. By age 65, women’s risk of developing AD is 1 in 5. Women also make up the majority of dementia carers. Therefore, older women may be particularly vulnerable to dementia‐related fear and its negative impacts. Across two studies, we examined (1) associations between dementia‐related fear and psychosocial functioning and (2) the effectiveness of a brief intervention to reduce fear in older women with and without a dementia family history.

**Method:**

Participants were women aged >55 years without a dementia diagnosis. In study 1, participants completed measures of dementia‐related fear and memory failures. In study 2, participants were randomized into intervention (psychoeducation, mindful grounding, exposure/behavioral activation) or control groups (psychoeducation, mindful grounding), and completed measures of dementia‐related fear, memory failures, depression, social function, and well‐being.

**Result:**

Study 1 included 285 women (66.1±7.1 years; Fx = 45.6%). Dementia‐related fear was positively associated with memory failures (*r* = .64, *p*<.001); fear was elevated in women with a dementia family history (*p* = .002, η^2^ = .03; Fig. 1A). Study 2 included 58 women (64.6±6.0 years; Fx = 55.2%) randomized to intervention or control groups. Again, dementia‐related fear was associated with memory failures (*r* = .59, *p*<.001); fear was elevated in women with a dementia family history (*p* = .013, η^2^ = .02). Fear was also associated with depression (*r* = .52, *p*<.001), and lower well‐being and social participation (*r*s>‐.59, *p*s<.001). Fear effects on depression and social participation were stronger for women with a dementia family history (*p*s<.027, η^2^s = .11). Fear scores decreased from pre‐ to post‐intervention (*p*<.001, η^2^ = .06). The biggest reduction was observed for women in the intervention group with a dementia family history (*p* = .004, η^2^ = .03; Fig. 1B).

**Conclusion:**

Older women with a family history of dementia are particularly fearful, and these fears are inversely related to mood, perceived memory functioning, social participation, and well‐being. A low‐cost, low‐intensity intervention can reduce dementia‐related fear and may be particularly beneficial for older women with a dementia family history. Targeting dementia‐related fear in women has important implications in the era of biomarker disclosure and direct‐to‐consumer genetic testing.